# Supplementary material for: Influence of Hydrophobicity of Backbone Polymer in Thermo-Responsive Hydrogel with Immobilized Amine on Cycle Capacity for Absorption and Recovery of CO2
Source: Polymers (Basel). 2019 Jun 10;11(6):1024. doi: 10.3390/polym11061024 (PMC6631255; doi:10.3390/polym11061024)
Supplement: Supplementary file 1 [file polymers-11-01024-s001.pdf]

Supporting Material

Influence of Hydrophobicity of Backbone Polymer in Thermo-responsive Hydrogel with Immobilized Amine on Cycle Capacity for Absorption and Recovery of CO_2

Yuma Nagasawa [1], Yoshimi Seida [2,*], Takehiko Gotoh [3] and Eiji Furuya [1]

1 Department of Applied Chemistry, Meiji University, 214–8571 Kawasaki, Japan; n7y2u6@gmail.com (Y.N.); egfuruya@meiji.ac.jp (E.F.)
2 Natural Science Laboratory, Toyo University, 112–8606 Tokyo, Japan
3 Department of Chemical Engineering, Graduate School of Engineering, Hiroshima University, 739–8527 Higashi Hiroshima, Japan; tgoto@hiroshima-u.ac.jp
* Correspondence: seida@toyo.jp; Tel.: +81–3-3945-4894

The CO_2 recovery performance of the gel system was roughly estimated referring to the process conditions of the existing process. The operating conditions of the gel system were determined based on the results obtained in this study. Using the adsorption model for the gel system constructed in this study, the amount of adsorbed CO_2 at each operating temperature was calculated. With reference to the cycle capacity obtained in this study, the $pK_{a,g}$ at desorption temperature was determined. The heat requirement was determined based on the heat capacity of water in the slurry because heating and cooling water is the major energy consumption. In the calculation of adsorption amount in the gel system, the composition of gel No.12 was used. The swelling ratio of the gel was set to 1/5 of gel No.12. In the case of a solid adsorbent, moisture in the air is also adsorbed in the CO_2 adsorption process, and therefore, the latent heat of the adsorbed water is also required at the time of CO_2 desorption and regeneration of the adsorbent. Water is also recovered during the desorption process in the gel system, but the latent heat will be suppressed because it is an amount of water vapor corresponding to the saturated vapor pressure in the reactor. (ex. p_{CO2}: p_{H2O} = 0.75:0.25 at 343 K). The consumption of sensible heat for heating and cooling the entire slurry in the gel system increases depending on the water content of the slurry.

Table S1 Performance comparison

Parameters		
Process	Temperature swing Gel system	Temperature- vacuum swing adsorption cycles [1]
Reactor Type	Gas-Liquid contact reactor	Gas-Solid contact reactor
Reactor Volume	50	15.7
Active material [kg/rector]	Thermoresponsive hydrogel with immobilized amine (3.5mmol amine/g gel)	Amine-functionalized polymer resin

	10	30
Number of reactor	8	8
Cycle number per day	4	1
Heating requirement [K]	343	353
Cooling requirement [K]	293	288
Heat requirement [MJ/kg]	105.9	27.3
Electricical requirement[MJ/kg]	no data	19.5
Feed CO_2 [ppm]	400	470
CO_2 production [kg/d]	2.53	2.8
Specific energy [MJ/kg-CO_2]	105.9<	46.8

1. Bajamundi, C.J.E.; Koponen, J.; Ruuskanen, V.; Elfving, J.; Kosonen, A.; Kauppinen, J.; Ahola, J. Capturing CO_2 from air: Technical performance and process control improvement. *J. CO_2 Util.* **2019**, *30*, 232–239, doi:10.1016/j.jcou.2019.02.002.
